# Evaluation of the impact of a psycho-educational intervention for people diagnosed with schizophrenia and their primary caregivers in Jordan: a randomized controlled trial

**DOI:** 10.1186/s12888-015-0444-7

**Published:** 2015-04-08

**Authors:** Abd Alhadi Hasan, Patrick Callaghan, Joanne S Lymn

**Affiliations:** School of Health Sciences, University of Nottingham, Queen’s Medical Centre, Nottingham, NG7 2UH UK

**Keywords:** Schizophrenia, Schizoaffective, Primary caregivers, Randomized controlled trial, Psycho-education

## Abstract

**Background:**

Psycho-educational interventions for people diagnosed with schizophrenia (PDwS) and their primary caregivers appear promising, however, the majority of trials have significant methodological shortcomings. There is little known about the effects of these interventions delivered in a booklet format in resource-poor countries.

**Methods:**

A randomized controlled trial was conducted from September, 2012 to July, 2013 with 121 dyads of PDwS and their primary caregivers. Participants aged 18 years or older with DSM-IV schizophrenia or schizoaffective disorder, and their primary caregivers, from four outpatient mental health clinics in Jordan, were randomly assigned to receive 12 weeks of a booklet form of psycho-education, with follow-up phone calls, and treatment as usual [TAU] (intervention, n = 58), or TAU (control, n = 63). Participants were assessed at baseline, immediately post-intervention (post-treatment1) and at three months follow-up. The primary outcome measure was change in knowledge of schizophrenia. Secondary outcomes for PDwS were psychiatric symptoms and relapse rate, with hospitalization or medication (number of episodes of increasing antipsychotic dosage), and for primary caregivers were burden of care and quality of life.

**Results:**

PDwS in the intervention group experienced greater improvement in knowledge scores (4.9 vs −0.5; p <0.001) at post-treatment and (6.5 vs −0.7; p <0.001) at three month-follow-up, greater reduction in symptom severity (−26.1 vs 2.5; p <0.001: −36.2 vs −4.9; p <0.001, at follow-up times respectively. Relapse rate with hospitalization was reduced significantly at both follow-up times in the intervention group (p <0.001), and relapse with medication increased in the intervention group at both follow-up times (p <0.001). Similarly there was a significant improvement in the primary caregivers knowledge score at post-treatment (6.3 vs −0.4; P < 0.001) and three month-follow-up (7.3 vs −0.7; p <0.001). Primary caregivers burden of care was significantly reduced in the intervention group (−6.4 vs 1.5; p <0.001; −9.4 vs 0.8; p <0.001), and their quality of life improved (9.2 vs −1.6; p = 0.01; 17.1 vs −5.3; p <0.001) at post-treatment and three month-follow-up.

**Conclusions:**

Psycho-education and TAU was more effective than TAU alone at improving participants’ knowledge and psychological outcomes.

**Trial registration:**

Current Controlled Trials ISRCTN78084871.

## Background

The health system in Jordan has three sectors: Ministry of Health (MoH), private and military. The MoH provides healthcare to the majority of the Jordanian population [1]. In Jordan, 305 individuals per 100,000 of the population have been diagnosed with mental illness, 50% of whom are diagnosed with schizophrenia [1].

Schizophrenia is one of the most common and serious forms of mental illness and is often chronic, recurrent, disabling and debilitating [2]. Previous studies have estimated that schizophrenia affects around 1.1% of the adult population worldwide, which equates to around 51 million people. Commonly, people are diagnosed with schizophrenia before the age of 25 years [3].

While studies have reported that the main cause of schizophrenia is unknown, a widely accepted model is the stress vulnerability hypothesis, which proposes that the interaction between biological vulnerability and socio-environmental stressors, including social stressors, have a significant role in the presentation and illness course [4]. This model suggests that schizophrenia is caused by an imbalance in biological and psychological systems. With an imbalance in biological systems, including genetics, head injury and viral infection, being considered a precipitating cause for schizophrenia. The impact of schizophrenia is commonly mitigated by taking medication and abstaining from alcohol [5]. The psychological system is concerned with stress; life events cause stress that often overwhelm people and compel them to adapt differently to stressful situations in order to function ‘normally’ [6,7]. However, people who struggle to adapt to stressful life events (e.g. bereavement, loss of job) often report poorer disease symptoms [5].

Psycho-educational interventions described in previous randomized controlled trials (RCTs) [8-10] sought to improve people diagnosed with schizophrenia (PDwS) and primary caregivers’ knowledge of schizophrenia, and to change their approach to dealing with disease symptoms using strategies described by these interventions [11]. Whilst the content of psycho-educational interventions varies between studies, common factors include general information about schizophrenia, symptoms, medication management, problem-solving strategies and communication skills for PDwS and primary caregivers [9,12-14]. Psycho-educational interventions have previously been delivered by psychiatrists [15] mental health nurses [8,9,15] and social workers [16]. The average duration of sessions varied among studies ranging from 60 to 120 minutes [9,12,17-19]. The methods of delivering psycho-educational interventions in studies for PDwS and primary caregivers include lectures [9,12,20-22], face to face methods, supported with a printed booklet [12,15] and online education [22].

Studies which adopted an online method of delivering psycho-educational interventions to participants revealed a substantial improvement in PDwS and family caregivers’ knowledge levels and psychiatric symptoms [10], stress and social support levels [22]. Additionally, delivering psycho-educational interventions with minimal interaction such as printed booklets has shown a similar effect on participants’ outcomes [22]. A recent meta-analysis of RCTs reported that psycho-educational interventions delivered online, by email or by printed leaflets were easy to access for large numbers of mental health patients and their primary caregivers at a relatively low cost. There has been an increasing interest recently in delivering psycho-educational interventions using less demanding and intrusive methods in relatively resource-poor countries [23].

Studies have shown that psycho-educational interventions may improve PDwS and primary caregivers’ outcomes, but many of the published RCTs have significant methodological shortcomings which limit the comparability of studies and weaken the validity of the conclusions drawn about their effectiveness. Some of the specific methodological flaws are associated with lack of adequate reporting of randomization, inadequate sample sizes to detect real differences in outcomes, high attrition rates and lack of blinding in assessments [24]. Consequently, the evidence base is inconclusive about the effectiveness of such interventions on PDwS and primary caregivers’ outcomes, hence the current study [25]. The main aim of this study was to investigate the effectiveness of a psycho-educational intervention delivered via a printed booklet with regard to PDwS and primary caregiver’s outcomes. The primary outcome was knowledge of schizophrenia. Secondary outcomes for PDwS were psychiatric symptoms and relapse rates and for primary caregivers, burden of care and quality of life at post-treatment and three-month follow-up.

## Methods

### Study design

A single-blind, randomized controlled trial to compare TAU alone with TAU and a psycho-educational intervention comprising six booklets delivered fortnightly to participants alongside follow-up phone calls.

### Participants

A total of 121 participants were recruited by the primary researcher and nurses between September, 2012, and July, 2013, in four mental health outpatient clinics in Amman, Jordan (Amman Consultant Clinic; National Centre for Mental Health (NCMH); Al-Hashmi Clinic; AL Bashir mental clinic).

Eligibility criteria were adults aged 18 or over diagnosed with schizophrenia or schizoaffective disorder according to the Diagnostic and Statistical Manual of Mental Disorders, 4^th^ Edition (DSM-IV) [26]. The diagnosis for the study purpose was taken from the PDwS clinical records at the outpatient clinic. The original diagnosis was made following a structured interview between a psychiatrist and the PDwS with family caregivers present, and recorded. Primary caregivers were those more involved in caring for their relative diagnosed with schizophrenia or schizoaffective disorder. All participants had to be able to read and write English or Arabic and be willing and able to consent.

Exclusion criteria were: People diagnosed with schizophrenia who had a learning disability, with known organic mental disorder, substance abuse, lived alone or without close contact with caregivers. PDwS currently receiving any formal psycho-educational intervention were also excluded. Primary caregivers involved in caring for more than one person diagnosed with mental health problems were excluded from the study.

The study was approved by the University of Nottingham Faculty of Medicine and Health Sciences Research Ethics Committee (Ref SNMP 12072012) and the Scientific Research Ethics Committee of the Ministry of Health, Jordan (Ref 9067). Written consent was obtained from all participants.

### Procedure

#### Randomization and masking

After baseline measurements, participants, who met the inclusion criteria, were randomly allocated to one of the study arms by a third person remote allocation system. The allocation of participants to the study arm was determined by a random number list generated by another researcher who had had no contact with, or access to, recruited participants. PC generated and sent a random list to the independent researcher; the primary researcher (AH) contacted the independent researcher when each participant was recruited. Outcome assessments (post-treatment & three month follow-up) were made by an independent researcher masked to the participants’ allocation. The allocation sequence was concealed until participants were assigned to either arm of the study, but the researcher and participants were not blinded to allocation thereafter.

Booklets were distributed in sealed envelopes to minimize contamination and protect participants’ anonymity. All booklets were kept with AH to avoid dissemination to other clinics or PDwS allocated to the control group. Participants receiving psycho-education and treatment as usual (TAU) were instructed not to share information with other PDwS and/or primary caregivers.

### Description of the control group

All four clinics are state funded and the care provided in these clinics was similar. All the participants in the study received treatment as usual consisting of medication, and laboratory investigations delivered by the mental health team.

### Therapy

PDwS in Jordan typically visit outpatient clinics with their family member. The study recruited people experiencing acute or long term symptoms being treated in these clinics when they attended for appointments.

Participants in the intervention group received treatment as usual, supported with psycho-educational booklets each fortnight for 12 weeks. Follow-up phone calls to primary caregivers were also made to ensure that they had read and understood the booklet and to allow them to ask questions about its content. The psycho-educational intervention was based on the framework of Atkinson and Coia [27] and its details are shown in Table 1.

**Table 1:** **The content of psycho-educational intervention**

**Booklet number**	**Goals**	**Contents**
One	To understand the nature of schizophrenia and its symptoms	- Diagnosis of Schizophrenia according to DSM-IV.
		- Truths and myths about schizophrenia
		- Symptoms of schizophrenia.
Two	To understand the causes of schizophrenia and the importance of the family in supporting affected individuals.	- Causes of schizophrenia
		- Stress vulnerability model
		- Role of the family.
Three	To improve participants understanding of antipsychotic medications and improve medication compliance	- Side effects of medications
		- Mechanism of action of medications
Four	To review relapse triggers & warning signs and improve participants ability to recognise these.	- Early warning signs of relapse
		- Common relapse triggers
		- Relapse management strategies.
		- Burden of care
Five	To improve understanding of problem solving interventions in schizophrenia.	- Problem solving interventions in schizophrenia.
		- Practical advice for problem solving
Six	To identify stress triggers and improve stress management techniques.	- Stress management skills and strategies.

The final versions of these booklets were reviewed and approved by a Professor of Psychiatry in the UK, independent of the study. Thereafter, three psychiatrists, four mental health nurses and six participants from the target population of the study were asked to assess the booklets in terms of their content, clarity and practicality. A comparison between treatment as usual and the psycho-educational intervention is shown in Table 2.

**Table 2:** **Comparison between treatment as usual and psycho-education intervention**

**Aspect**	**Treatment as usual**	**Psycho-education intervention**
General description	Medication prescription, lab investigation and limited explanation by mental health team providers for some questions.	Treatment as usual supported with psycho-educational booklets.
Form	Verbal over short time	Six psycho-educational booklets with follow-up phone calls to ensure that they have read and understood the booklet and to allow them to ask questions about its content.
Key content	Participants question (unspecified)	Each booklet discussed the different topic. Booklet one & two focused on illness general information. Booklet three outlined medications and side effect. Booklet four explained relapse warning signs and prevention. Booklet five mentioned problem-solving techniques and booklet six illustrated some skills to cope with illness symptoms.
Use of written	None	Simple and well-designed booklet.
Mode of delivery	Mental health providers	Primary researcher.
Timing	On day of visiting psychiatric clinic	Each fortnight.

Booklets were printed in the form of a double side A4 page in colour. The research team created the booklet in a short, simple format for ease of reading especially to those with poor concentration and short attention spans. In addition, Tables and Figures were deployed to improve clarity and understanding. The content of each booklet included information on diagnosis, myths about schizophrenia, symptoms, coping with symptoms, treatment options and how to live better with schizophrenia and have meaningful and satisfying lives.

### Measures

The primary outcomes for PDwS and primary caregivers were knowledge of schizophrenia measured by the Knowledge about Schizophrenia Questionnaire (KASQ). KASQ is a self-report questionnaire containing 25 items measuring participants’ knowledge of schizophrenia and its management, aetiology, prevalence, prognosis and treatment It is scored from 0 to 25 with a higher score indicating more knowledge, has Cronbach’s alpha coefficients of between 0.85 – 0.89 and a test-retest reliability coefficient over three weeks of 0.83 [28]. An Arabic version of the KASQ used in this study had high content validity by expert review and excellent reliability (Cronbach’s alpha, 0.88).

Secondary outcomes were schizophrenia symptoms measured by the Positive and Negative Symptom Scale (PANSS) for PDwS, Family Burden of Care measured by the Family Burden Interview Scale (FBIS) and quality of life measured by the Schizophrenic Carers’ Quality of Life Scale (S-CQoL), for primary caregivers. PANSS measures 30 clinical symptoms of schizophrenia; each symptom is scored from 1 indicating absence of psychopathology to 7 indicating severe psychopathology, with higher scores indicating poorer mental health status. Internal reliability and criterion-related validity are 0.77 (positive scale) and 0.77 (negative scale), and 0.52 with the Clinical Global Impression scale (CGI) [29]. The primary researcher (AH) attended training delivered by the PANSS Institute, USA, and trained the outcomes assessors. An inter-rater reliability, checked prior to the study, between assessors was 0.75 and inter-rater reliability (intra-class correlation (ICC) was 0.79. This tool was administered in English by the primary researcher (AH) and research assistants.

The FBIS has 24 items and focuses on six domains of primary caregivers’ burden: family finance, routine, leisure time, physical health, mental health and family interaction. Each item is rated on a three-point Likert scale (0: no burden, 1: moderate burden, 2: severe burden) scored from 0 to 48; a higher score indicates a higher level of burden. The scale has a Cronbach’s alpha of 0.87 and test-retest reliability of 0.83 [30]. The translated version showed excellent reliability (Cronbach’s alpha, 0.86) and inter-rater reliability (ICC, 0.86).

The S-CQoL has 25 items measuring seven dimensions: Physical and Psychological Wellbeing (PsPhW), Psychological Burden and Daily Life (PsBDL), Relationships with Spouse (RS), Relationships with Psychiatric Team (RPT), Relationship with Family (RFa), Relationships with friends (RFr) and Material Burden (MB), total score ranged from 25–125, a higher score indicates a better quality of life. Cronbach’s alpha is 0.79 to 0.92 [31]. The Arabic version demonstrated excellent internal consistency (Cronbach’s alpha, 0.87) and inter-rater reliability (ICC, 0.87).

Relapse was defined by hospitalization (the number of readmissions three months prior to the study commencing, immediately post intervention and at three months follow-up) and the number and dosage of antipsychotic medications prescribed to participants during the same intervals. Inter-rater reliability (Kappa agreement) was 0.43.

As none of the measures had been used in an Arabic speaking country previously, they were translated from English to Arabic, back translated to English and checked for discrepancies by an independent bilingual translator and the original author. A pilot study with two PDwS and two primary caregivers confirmed participants’ acceptability and understanding of the scales.

### Analysis

#### Sample size

The sample size was estimated based on previous research which showed a change in the knowledge score of 2 points post-treatment [11,32]. Taking into consideration a power of 80% and significance level of p < 0.05, allowing for 15% attrition, deduced from previous studies, we estimated 144 participants would be required.

### Statistical analysis

All data were analyzed by using SPSS version 21. Analysis was done by intention to treat with the last observation carried forward to handle missing data at post-treatment and three-month follow-up. Demographic data were summarized by frequencies and percentages. A Goodness of Fit Chi-square test was employed for categorical variables and Independent samples *t*-test were used for continuous variables. The mean scores between groups on all outcome measures were compared using an independent sample *t*-test or chi-square, as appropriate. To control for type I errors for multi-comparison tests, Bonferroni’s adjustment was used to adjust the level of significance set at baseline for all statistical tests to the 1% level (p < 0.01). Analysis of variance (between and within) was used to determine whether treatment produced between and within group and interactive effects of treatment by time for each outcome. The McNemar test was used to identify the difference in relapse rates between groups from baseline, post-treatment and at three month follow-up.

## Results

**Figure 1:**
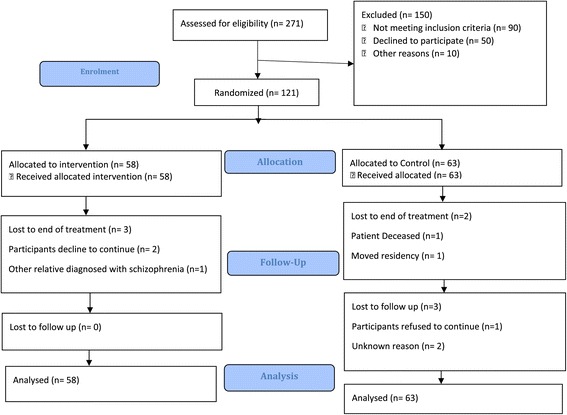
**Trial profile.**

One hundred and twenty-one PDwS/primary caregiver dyads provided consent and were randomly allocated to psycho-education and TAU (n = 58) or TAU (n = 63) (Figure 1). Baseline characteristics of participants are shown in Table 3. There was no statistically significant difference between the groups on baseline characteristics at the 1% level of significance, (adjusted P value for the type I error protection).

**Table 3:** **Baseline characteristics of people diagnosed with schizophrenia and primary caregivers**

**Characteristics**	**Interventional group (n = 58)** ^₮^		**Control group (n = 63)** ^**¥**^	
	**Frequency**	**%**	**Frequency**	**%**
Patients				
Age, years (M, SD)	(40.4, 8.6)		(41.1, 7.9)	
≤20	2	3°4	0	0.0
21-30	7	12.1	7	11.0
31-40	25	43.1	22	35.0
41-50	19	32.8	24	38.1
≥50	5	8.6	10	15.9
Gender				
Male	38	65.5	46	73.0
Female	20	34.5	17	27.0
Education level				
Primary school or below	18	31.0	22	35.0
Secondary school	22	37.9	27	42.8
College or above	18	31.1	14	22.2
Employment status				
Employed	12	20.7	18	28.6
Unemployed	42	72.4	35	55.5
Other	4	6.9	10	15.9
Marital status				
Married	23	39.7	30	47.6
Single	31	53.4	29	46.0
Divorced	4	6.9	3	4.8
Other	0	0.0	1	1.6
Illness duration at baseline in years (M, SD)	12.2 years (9.3)		12.8 years (9.00)	
≤2	6	10.3	9	14.3
3-5	12	20.7	10	15.9
≥5	40	69.0	44	69.8
Diagnosis				
Schizophrenia	32	55.2	33	52.4
Schizoaffective	26	44.8	30	47.6
Primary caregivers				
Age, years (M, SD)				
≤20	(47.05, 10.6)		(50.4, 12.7)	
21-30	0	0.0	0	0.0
31-40	2	3.4	2	3.2
41-50	16	27.6	15	23.8
≥50	21	36.2	18	28.6
	19	32.8	28	44.4
Gender				
Male	10	17.2	19	30.2
Female	48	82.8	44	69.8
Employment status				
Employed	18	31.0	13	20.7
Unemployed	34	58.6	42	66.6
Retired	6	10.3	8	12.7
Marital status				
Married	47	81.0	54	84.1
Single	6	10.3	6	9.5
Divorced	1	1.7	0	0.0
Other	4	7.0	3	4.8
Relationship to patient				
Parents	24	41.3	24	38.1
Sibling	10	17.2	20	31.7
Spouse	20	34.5	19	30.2
Child	4	7.0	0	0.0
Monthly income				
≤100	5	8.6	4	6.3
101-300	29	50.0	40	63.5
301-600	18	31.1	14	22.3
≥601	6	10.3	5	7.9

M: Mean; SD, standard deviation in parentheses; ^₮^(structured psycho-educational intervention) group; ^¥^Control group (treatment as usual - standard outpatient care); JoD = 1.4 $ US.

### Intervention effect on the people diagnosed with schizophrenia outcomes

#### Knowledge of schizophrenia and psychiatric symptoms

An exploration analysis performed on dependent variables at pre-test and two post-tests to examine preliminary assumption for mixed between-within subject ANOVA on tests of normality, linearity, multi-collinearity, univariate and multivariate outliers and homogeneity of variance revealed no serious violation to test assumptions [33].

Data from the primary outcome of the PDwS showed there were no statistically significant differences in KASQ and PANSS scores at baseline between two groups (Table 4).

**Table 4:** **KASQ & PANSS and FBIS with S-CQoL scores at pre-test & post-tests and result for repeat measure ANOVA test (Group x Time) between the intervention and control group**

	**Interventional group (n = 58)** ^₮^						**Control group (n = 63)** ^**¥**^						**Repeat measure ANOVA**		
	**Pre-test**		**Post-treatment**		**Three-month follow-up**		**Pre-test**		**Post-treatment**		**Three-month follow-up**		**Time X Group**	**Time**	**Group**
**Instrument**	**M**	**SD**	**M**	**SD**	**M**	**SD**	**M**	**SD**	**M**	**SD**	**M**	**SD**	**F**	**F**	**F**
KASQ (0–25)ª	7.97	2.96	12.95	3.02	14.50	3.02	8.13	3.25	7.59	3.16	7.48	3.39	193.82***	128.85***	59.61***
PANSS (30–210)	97.22	13.01	71.01	14.32	61.00	14.43	92.27	20.54	94.79	22.54	87.38	21.16	75.06***	105.72 ***	27.29***
Primary caregivers															
KASQ (0–25)ª	9.45	4.30	15.71	3.41	16.74	3.28	8.22	3.82	7.80	3.67	7.51	3.68	186.55***	131.30***	96.31***
FBIS (0–48)	28.26	7.22	21.86	6.67	18.84	6.63	25.44	8.32	26.98	8.66	26.22	8.33	73.94***	48.36 ***	6.08*
QOL(1–125)	59.93	16.23	69.16	15.04	77.07	14.64	63.49	15.64	61.87	16.66	58.19	15.93	75.98***	21.70***	8.02**

Note: ^₮^Interventional (structured psycho-educational intervention) group; ^¥^Control group (standard outpatient care); M, Mean; SD, Standard Deviation; KASQ, Knowledge About schizophrenia Questionnaires; PANSS, Positive and Negative Syndrome Scale.

Pre-test = Baseline measurement before the start of intervention; Post-treatment = immediately after intervention; second follow-up = 3 months after intervention.

ªPossible range of scores of each scale indicated in parenthesis; Possible range of scores of each scale indicated in parenthesis.

***p < 0.001, **p < 0.01, *p < .05.

In comparison with those in the control group, participants in the intervention group had statistically significant improvements in KASQ scores at post-treatment and three-month follow-up. Mauchly’s test of spherecity was significant (p <0.05), and hence a Greenhouse Geisser correction for the df value was performed [34]. Interaction between group by time was significant for KASQ (p <0.001, univariate eta squared =0.62 (large effect) [35] and significant time effect was observed for KASQ (p < 0.001, univariate eta squared = 0.52 (Large effect). In addition, the result demonstrated a significant group effect (treatment) on KASQ (p <0.001, univariate eta squared = 0.33 (large effect). This shows an improvement in the knowledge level over the follow-up period in the intervention group.

With regard to PANSS scores, there was a significant interaction between group and time (p < 0.001, univariate eta squared = 0.39 (large effect) and significant effect time found on PANSS scores (p < 0.001, univariate eta squared = 0.47 (Large effect). The findings also showed a significant difference in terms of the group effect (p <0.001, univariate eta squared = 0.19 (Large effect). These results show that receiving the psycho-education intervention was associated with a reduction in symptom severity at post-treatment and three-month follow-up.

### Relapse

McNemar tests showed that, of the 58 PDwS allocated to the intervention group, 3 (5.2%) had relapsed, measured by hospitalisation, at post-treatment and 4 (6.9%) at three month follow-up compared with 31 (49.2%) and 32 (50.8%) respectively in the control group. Medication use was higher in the intervention arm 21 (36.2%) and 14 (24.1%) at post-treatment and three month follow-up, compared with 15 (23°8%) and 5 (7.9%) in the control arm at the same intervals. Data relating to an increment in antipsychotic dosage was reported directly from clinical records. Table 5 shows the time effect on relapse rate between the intervention and control groups.

**Table 5:** **Relapse rates of intervention and control groups**

	**Pre-test**				**Post-treatment**				**Three-month follow-up**			
	**Relapse H**		**Relapse M**		**Relapse H**		**Relapse M**		**Relapse H**		**Relapse M**	
	**N**	**(%)**	**N**	**(%)**	**N**	**(%)**	**N**	**(%)**	**N**	**(%)**	**N**	**(%)**
**Intervention group (n = 58)** ^₮^	23	(39.7%)	29	(50.0%)	3	(5.2%)	21	(36.2%)	4	(6.9%)	14	(24.1%)
**Control group (n = 63)** ^**¥**^	36	(57.1%)	20	(31.7%)	31	(49.2%)	15	(23.8%)	32	(50.8%)	5	(7.9%)
P values	P = 0.67		P = 0.13		P < 0.001***		P = 0.002**		P < 0.001***		P < 0.001***	

Note: ^₮^Interventional (structured psycho-educational intervention) group; ^¥^Control group (standard outpatient care); Relapse H, Relapse with Hospitalization; Relapse M, Relapse with Medication.

Pre-test = Baseline measurement before the start of intervention; Post-treatment = immediately after intervention; second follow-up = 3 months after intervention.

***p < 0.001, **p < 0.01.

Number of relapse with admission to a psychiatric hospital at baseline (pre-test) and both post-tests; Number of relapse with increasing anti-psychotic medication dosage at baseline (pre-test) and both post-tests.

### Intervention effect on the primary caregivers’ outcomes

There were no statistically significant differences between the intervention and the control groups on baseline measures. Mauchly’s test of spherecity was significant (p <0°05), and hence a Greenhouse Geisser correction, for the df value was performed [34]. The interaction between groups by time was significant for KSQ, FBIS and S-CQOL scores. Moreover, the group and time effect were statistically significant for all primary caregiver outcomes. This illuminates the positive impact of the psycho-educational intervention on all primary caregiver’s outcomes over different follow-up times (Table 3).

## Discussion

To our knowledge, this is the first randomized control trial using psycho-education in the described format for PDwS and their primary caregivers. It is also the first such trial conducted in an Arab-speaking country.

In terms of PDwS the improvement in knowledge scores seen following the intervention corroborates previous reports which showed similar effects, albeit with a different population and intervention [10,11]. The finding of primary caregivers’ knowledge scores is inconsistent with those of other authors who reported that the intervention effect on the family member is not sustainable following the intervention. In the current study whilst knowledge scores did improve significantly at three-month follow-up compared with post-treatment among primary caregivers, this was the case for PDwS who showed a further significant increase in knowledge at 3-months follow-up compared to post-treatment. This may be attributed to written material having the advantage of being available to refresh participants’ memory as needed and accessing information at their own convenience. It is noteworthy that there no difference at 3-months follow-up compared to post-treatment among primary caregivers. However, PDwS scores demonstrated further improvement over the same interval. This may be linked to the fact that primary caregivers were able to absorb and assimilate the information more quickly when compared with their mentally ill relative who may have needed more time to consolidate their understanding of the material.

Our findings confirm that adding a brief psycho-educational intervention to routine care in a psychiatric clinic is an effective way to ameliorate significant symptoms of schizophrenia. Whilst findings from previous studies about schizophrenia symptomatology are inconsistent, most trials have shown that the severity of psychiatric symptoms can be reduced post-treatment and at follow-up [2,11]. These findings are due possibly to improved knowledge about symptoms and a better understanding of anti-psychotic medication impacting positively on people’s mental health. The booklet method used in this study afforded participants an opportunity to re-read the information at their own leisure and this may have enabled people to tailor the information to their own needs. Another possible explanation is that we engaged primary caregivers who lived with people diagnosed with schizophrenia and supervised them when they used anti-psychotic medication.

In accordance with previous findings, there was a significant difference between the two groups in relapse rate as measured by readmission rates and medication use. However, one unanticipated finding was the significant increase in medication rates in the intervention group at post-treatment and three-month follow-up when compared with the control group. The content of the psycho-educational intervention included a booklet about early warning signs of relapse which might have allowed participants to take immediate action in terms of medication use, if these symptoms occurred. The finding of this study clearly affirms the positive effects of such interventions as they are designed to improve participants’ awareness about illness, improve their communication and problem solving skills in everyday situations, reduce emotional over-involvement as well as increased their adherence with antipsychotic medication, resulting in changing relapse rates between groups [15,16,25]. Also, teaching primary caregivers about anti-psychotic medication may have led to them supervising their relative with schizophrenia when he/she used medication [36]. Moreover, the psycho-educational intervention offered a combination of information covering cognitive, psychomotor and behavioral components to change attitudes. Relapse rate reduced in both groups at both follow-up points, however, it was statistically significant favoring the intervention group. Overall, this reduction in both groups might be explained potentially by the fact that the number of psychiatric beds in Jordan’s mental hospital is limited to 8.27 beds per 100,000 population [1].

In the light of secondary outcomes for primary caregivers’ (burden of care and quality of life), the study findings showed a significant change in all outcomes in the intervention group at post-treatment and three month follow-up. Greater reduction in family burden of care scores baseline to post-treatment and three month follow-up compared with the control group was attributed to their participation in the psycho-educational intervention. They may have gained new caregiving skills in coping with disruptive behaviour. In addition, they might have gained more confidence to deal with their relative’s behaviour. This is consistent with earlier studies about the positive effect of psycho-education interventions on family burden [9,12].

Our control findings revealed deterioration in most outcomes and a slight improvement in some outcomes. In other words, these findings suggest treatment as usual in the psychiatric outpatient clinics in this study did not meet the needs of people diagnosed with schizophrenia and their primary caregivers.

The Psychoeducational model adopted in this study suggests that improving people diagnosed with schizophrenia and primary caregivers’ knowledge about schizophrenia and its management improves the relationship between PDwS and primary caregiver with mental health professionals, and improves their confidence in dealing with ill relatives’ unexpected or challenging behaviour. Improving their insight may change their attitudes and reduce potential stigma. As a result, their burden of care may be reduced, and their quality of life improved [27]. However, it is worth noting that this improvement at three months follow-up has been demonstrated in previous studies, but we cannot be certain that the positive effects of the psycho-educational intervention would persist beyond this period without longer follow-up.

There are several limitations to this study. First, most of the outcome measures are self-report, and this could cause response bias. Second, we did not monitor medication compliance, but the differences between the control and interventions arms remained after using ANCOVA to control for the possible effect of increases in medication dosage on outcomes. This mitigates this limitation, but it does not exclude it. Psycho-educational interventions aimed at improving participants’ understanding of medication might have a significant effect on medication compliance, given that the level of medication compliance can produce a robust effect on participants’ outcomes. Thus, it may be that reductions in relapse rates or improvements in psychiatric symptoms were due to medication compliance. Thirdly, using this method of education we could not be sure that participants read the booklets from the trial data. However, the trial being reported was part of a larger mixed methods study that also included a process evaluation in which we used qualitative interviews to investigate participants’ experiences of the intervention. Data from these interviews show that participants in the trial reported that they had read the booklets and this concurs with the significant increases in their knowledge scores. The need to translate the measures used into Arabic may be considered as a possible limitation of the study, although no issues were identified following translation and back-translation of the measures.

Despite these limitations, our results are significant in several ways. The study added to the evidence about effectiveness of a novel format in delivering a psycho-educational intervention and was designed and conducted in accordance with the CONSORT statement guidance for trials of this nature [24]. Specifying primary and secondary outcomes prior to the study commencing minimized the likelihood of type I error. Recruitment occurred in four psychiatric clinics, and this increases the likelihood of a representative sample. The need for further research with longer follow-up is, however, evident. This will enable researchers to understand the sustainability of the intervention.

In the comprehensive Cochrane systematic review of family intervention for schizophrenia that was updated in 2011, no study conducted psycho-educational interventions in a resource-poor country such as Jordan [25]. Our findings are crucial because we have tested this intervention for the first time in a resource poor, low income country in terms of the intervention itself and the delivery method.

Although a large body of literature conducted in developed countries confirms the effectiveness of this approach in treating PDwS, most studies report low engagement rates due to social stigma, particularly in developing countries [9]. Therefore, the booklet method of applying these interventions provided a valuable solution to overcome the main barriers of previous studies: using evidence-based interventions that are cost-effective and acceptable to participants and their caregivers. In the Jordanian context, PDwS and their primary caregivers shared characteristics including low education levels, living together, poor knowledge about mental illness and low socio-economic status. The intervention was developed to address these issues.

We designed the intervention used in this study on adult learning theory the main tenets of which are enhancing and/or changing people’s knowledge, attitude and behavior and our result shows we succeeded in this endeavor.

Currently, mental health services in Jordan do not involve PDwS’ education in its treatment approaches, thus we recommend policymakers need to take our findings into account when planning and delivering services and integrate psycho-educational programs into routine treatment in all mental health clinics. The innovative method of delivering the intervention in this study can be used with little staff training and additional resources, and is relatively simple, accessible and generates positive outcomes for PDwS and their primary caregivers.

## Conclusions

As far as we are aware, our study is the first adequately powered, randomized controlled trial investigating psycho-education delivered via booklets, internationally and in Arab speaking countries, assessing participants’ knowledge of schizophrenia, and positive and negative symptoms, relapse and caregivers’ burden of care and quality of life. Our findings have added to existing literature using an intervention that is less intrusive with fewer demands than individual face to face or online methods. Furthermore, our findings suggest psycho-education delivered in this form is effective, acceptable, and relatively easy to design.
